# Investigation of the Proportion of *Brucella abortus* and *Brucella melitensis* in Sheep and Goat Milk

**DOI:** 10.1155/2023/6751152

**Published:** 2023-11-24

**Authors:** Saeid Rostami, Ehsan Rashidian, Amin Jaydari, Heidar Rahimi

**Affiliations:** ^1^Department of Pathobiology, Faculty of Veterinary Medicine, Lorestan University, Khoramabad, Iran; ^2^Department of Pathobiology, Faculty of Veterinary Medicine, Semnan University, Semnan, Iran

## Abstract

Despite the implementation of brucellosis eradication programs in Iran, this disease is still endemic and highly prevalent among ruminants in this country. The infection of small ruminants with *Brucella abortus* may play a significant role in the continuation of brucellosis among the herds of ruminants. This study investigated the proportion of *B. abortus* and *Brucella melitensis* in 150 samples of sheep and goat's raw milk which were obtained from Lorestan and Hamadan provinces in the western part of Iran using the PCR method. The results revealed that among the *Brucella* spp. positive samples, 26.5% and 73.4% of the samples were infected with *B. abortus* and *B. melitensis*, respectively. The incidence rates of *B. abortus* among the sheep and goats samples were 6.8% and 12.5%, respectively. There was a significant difference between goats and sheep regarding the proportion of *B. abortus*. Three samples (2%) (2 goats and one sheep) were simultaneously infected with both *B. melitensis* and *B. abortus*. This article renews our knowledge about the causative agent of brucellosis in small ruminants and shows that *B. abortus* has a relatively high prevalence among those animals in the western regions of Iran, and its role as one of the main factors of abortion among small ruminants should not be ignored. The information provided in the present study is important for the surveillance program, as eradication programs and strategies to prevent the spread of *B. abortus* among small ruminants that have not been vaccinated against this microorganism may be adapted accordingly.

## 1. Introduction

Brucellosis is a zoonotic disease that is considered a major public health concern worldwide [1]. In Iran, brucellosis is endemic and infects a broad range of animal species causing massive economic damages [2–4]. *Brucella melitensis* is the main cause of brucellosis in sheep and goats. Nonetheless, other species of *Brucella* can infect these animals [5]. Cows and buffaloes are the classic and preferred hosts for *Brucella abortus*. Few studies have reported the presence of *B. abortus* in sheep and goat herds from 1 to 4% [6, 7]. Although *B. abortus* infection in small ruminants is rare, a number of studies have confirmed that *B. abortus* is not prevalent among small ruminants [8].

Considering the existence of common pastures for livestock and the excessive movement of the herds of animals due to summer-winter migration in Iran, it is necessary to examine the rate of the transmission of *Brucella* isolates from a herd of livestock to the other herds and the presence of *Brucella* species in their nonspecific hosts in livestock farms, especially in the western areas of Iran. In these areas, brucellosis is very common and milk and dairy products, including the raw dairy products, play an important role in the preparation of local cheese in the diet of the people. Knowledge about the spread of brucellosis and its presence in different species of livestock is essential for adopting effective control measures. Therefore, this study aimed to investigate the proportion of *B. abortus* and *B. melitensis* in sheep and goat milk samples in Lorestan and Hamadan provinces in the western part of Iran.

## 2. Materials and Methods

### 2.1. Study Design and Research Area

**Figure 1:**
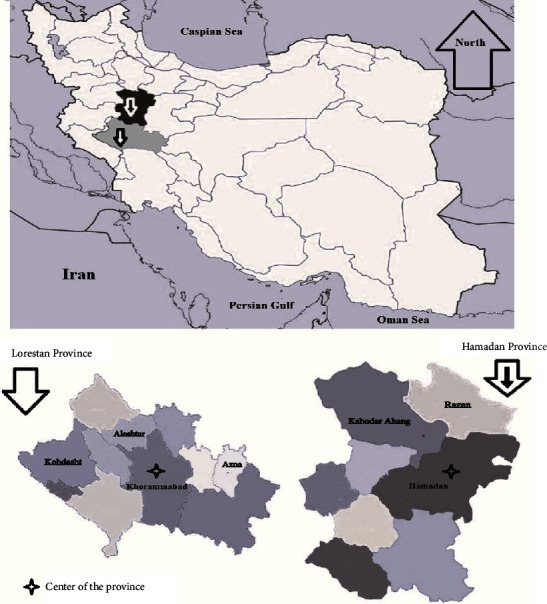
Map of Iran showing the geographic location of the study areas mentioned in article.

This study was conducted by determining the causative agent of brucellosis among sheep and goats in Lorestan and Hamedan provinces (western Iran) from March to July 2020. Lorestan province, with an area of about 28157 km^2^, is located in the southwestern region of Iran between 46° and 50ʹ to 50° and 1ʹ east longitude and 32° and 40ʹ to 34° and 23ʹ north latitude from the Greenwich meridian. This province with 800,000 hectares of good quality pastures and a population of about 4 million small ruminants (sheep and goats) ranks first in the livestock density index in Iran. For this purpose, 4 regions of the province were investigated. Also, Hamedan province with an area of 20172 km^2^, with a western position, is located between 59° and 33ʹ to 49° and 35ʹ north latitude and 34° and 47ʹ to 34° and 49ʹ east longitude from the Greenwich meridian. This province, with a population of 1.5 million small ruminants, is one of the provinces with high prevalence of Malta fever in the country, from which two regions with high livestock population were investigated (Figure 1).

### 2.2. Collection of Samples

In this study, 150 samples of raw milk (90 sheep, 60 goats) were collected from 6 regions of Lorestan (Khoramabad, Aleshtar, Azna, and Kohdasht) and Hamadan (Kabodar Ahang and Razan) provinces (Iran) from sheep and goats with a history of abortion in the sampling year. The areas that were selected for sampling had the highest prevalence of human brucellosis (0/025%–0.039%) in the studied province based on the report of the regional health department. More specifically, first, after disinfecting the nipples using 70% alcohol, 50 ml of milk was collected from each livestock. Second, information about the farm and the type of livestock was recorded. Finally, the milk samples along with ice were taken to the laboratory and were stored at −20°C until the molecular tests were performed.

### 2.3. DNA Extraction

After removing the milk samples from the freezer and thawing (at room temperature) them, first, 10 ml of each sample was centrifuged in 10 ml Falcons at 6000 rpm for 10 minutes. Next, 200 *μ*l of the fatty top layer was transferred to a 1.5 ml tube for performing DNA extraction using the Blood Genomic DNA Extraction Mini Kit (Favorgen, Taiwan) based on the protocol of the manufacturer. The concentration of extracted DNA was measured using a NanoDrop spectrophotometer (Thermo Scientific, Waltham, USA). Finally, the extracted DNA was stored at −20°C for carrying out the PCR procedure.

### 2.4. DNA Amplification and Detection of Polymerase Chain Reaction (PCR) Products

The polymerase chain reaction (PCR) method, explained by Baily et al. [9], was used to determine the genus *Brucella* spp. by identifying the *bcsp31* gene with a size of 223 bp. *B. melitensis* and *B. abortus* species were identified by detecting the IS711, repetitive genetic element, using specific primers for *B. melitensis* species (731 bp) and *B. abortus* species (498 bp) as previously described by Bricker and Halling [10].

PCR amplification was performed using the PCR master kit (Ampliqon Taq DNA Polymerase Master Mix RED 1.25 mL, Ampliqon Denmark) with 25 *μ*L mixtures which contained 12.5 *μ*L of 2X master mix, 0.5 *μ*L of each primer, and 4 *μ*L extracted DNA and 7.5 microliters deionized water. The Rev-1 and RB51 vaccine strains (supplied by the Iranian company named Razi Vaccine and Serum Research Institute) were used as the positive control for *B. melitensis* and *B. abortus,* respectively. For the negative control, sterile water was added instead of nucleic acids as the negative control. Furthermore, the amplification was performed using the Bio-Rad thermocycler (Model T-100, USA) under the following conditions: A for *Brucella* spp., B for *B. melitensis*, and C for *B. abortus*.

  A: The initial step of 95°C for 5 min, followed by 40 cycles of 90°C for 1 min, 60°C for 1 min, 72°C for 1 min, and finally, 72°C for 7 min.
  B: The initial step of 95°C for 5 min, followed by 35 cycles of 95°C for 1.15 min, 55.5°C for 2 min, 72°C for 2 min, and finally, 72°C for 5 min.
  C: The initial step of 95°C for 5 min, followed by 35 cycles of 95°C for 1.15 min, 55.5°C for 1 min, 72°C for 2 min, and finally, 72°C for 10 min.

The amplified products were separated in a 1.2% (w/v) agarose gel (Merck, Germany) which contained 2.5 *μ*g/mL gel stain (CinnaGene, Tehran, Iran). Electrophoresis was performed in 0.5x Tris/Borate/EDTA (TBE) buffer at 100 V for one hour. The results of gel electrophoresis were visualized under a UV transilluminator (E-Box, Iran). The 100 bp DNA ladder (Smobio, Taiwan) plus was used as the molecular size marker.

### 2.5. Statistical Analysis

The obtained data were analyzed by the chi-square test using SPSS Software Ver. 20 (SPSS Inc., Chicago, IL, USA). The *p* value <0.05 was considered significant.

## 3. Results

**Figure 2:**
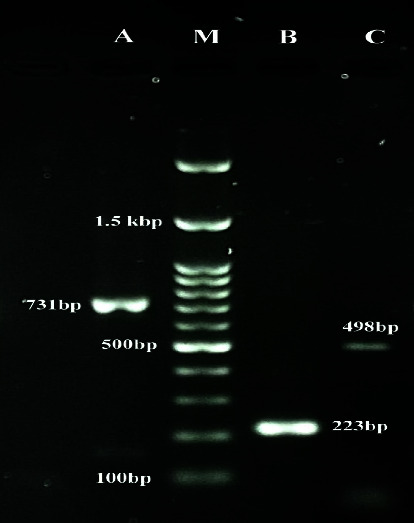
PCR assay for the detection of *Brucella*. Lane A: positive sample for *Brucella melitensis*; Lane M: standard DNA marker; Lane B: positive sample for *Brucella spp.*; Lane C: positive sample for *Brucella abortus*.

In this study, 150 milk samples were obtained from Lorestan and Hamadan provinces. PCR results showed that 49 of these milk samples (32.6%) had *Brucella* spp. infection; 36 (24%) and 13 (8.6%) samples out of all specimens (aborted animals) showed *B. melitensis* and *B. abortus* infection, respectively (Figure 2).

A total of 31 positive *Brucella* samples were obtained from sheep. Based on the results, 23.5% (24 samples) of these samples were infected with *B. melitensis* and 6.8% (7 samples) of them were infected with *B. abortus*. On the other hand, 18 positive *Brucella* samples were obtained from goats. On the basis of the results, 25% (12 samples) of these samples were infected with *B. melitensis* and 12.5% (6 samples) of them were infected with *B. abortus* (Table 1). In the present study, the prevalence of *B. abortus* in goat population was higher than that of sheep, so this difference was statistically significant (*p* < 0.05). The present study also indicates that *B. abortus* may play an important role in abortion in sheep and goats. It should be mentioned that the presence of *B. abortus* in the investigated population, all with a history of abortion, was 8.6%.

In Hamadan province, 18 positive samples of *Brucella* (36%) were identified. The results showed that 15 of the infected samples were infected with *B. melitensis* (30%) and 3 of them were infected with *B. abortus* (6%). No coinfections of *B. melitensis* and *B. abortus* in herds were observed.

In Lorestan province, 31 positive samples of *Brucella* (31%) were identified. Twenty-one of these samples were infected with *B. mellitensis* (21%), and 10 of them were infected with *B. abortus* (10%). Nonetheless, among these samples, 3 of livestock (3%), including 2 goats and 1 sheep, were simultaneously infected with *B. abortus* and *B. melitensis*. In the examined areas, 13 positive samples of sheep and goats were identified in Aleshtar city. Based on the results, 7 of these samples (28%) showed the highest rate of infection with *B. abortus* (Table 1).

## 4. Discussion

Brucellosis is endemic in most parts of Iran, and its annual incidence is still high among livestock and human populations. The prevalence of brucellosis in the western part of the country is higher than its other parts. More specifically, in Lorestan and Hamadan provinces, the average annual incidence of human brucellosis is estimated at 31 to 41 people per a population of 100,000 [11]. Meanwhile, despite the implementation of vaccination programs in these areas, brucellosis has not only not been controlled but has shown an increasing trend [2, 11] Therefore, in the present study, the proportion and host specificity of *B. abortus* strains were investigated in the milk samples obtained from sheep and goats with a history of abortion in the year of sampling in these areas. Based on the results, brucellosis could be a causative agent of 32.6% of abortion cases among these animals. Although the role of bacteria such as *Campylobacter*, *Chlamydia*, Q fever, and *Listeria* in abortion should not be ignored since these animals had a history of abortion for a few days or months before sampling, probably about 32.6% of the abortion cases in these animals can be caused by the genus *Brucella,* and the share of *B. abortus* is about 8.6%.

*Brucella* spp. were detected in the milk samples of 49 of the animals, of which 36 (73.4%) and 13 (26.5%) samples showed infection with *B. melitensis* and *B. abortus*, respectively. Out of the all *B. melitensis* infected milk samples, 24 (23.5%) and 12 (25.0%) cases were from sheep and goats, respectively. Also, 7 (22.5%) and 6 (33.3%) cases of milk specimens, infected with *B. abortus*, were related to sheep and goats, respectively.

The results of this study showed that *B. abortus* has a lower level in sheep population than goats. This issue may stem from the greater tendency of the goats to leave the herd and the possibility of their close contact with the cows on the farms. Moreover, it may result from the greater susceptibility of goats to the *B. abortus* species. In the study by Wareth et al. [6], there was a higher incidence of *B. abortus* species among the goats in comparison with the sheep. All ruminants are susceptible to *B. abortus*. Nonetheless, reports of the small ruminants which are infected with *B. abortus* are very rare [6, 7]. More specifically, few studies have reported a low rate of *B. abortus* in sheep in Iran [12], the United States [13], Nigeria [14], and Italy [7]. However, the present study showed a significant increase in the *B. abortus* infection in small ruminants in comparison with the previous reports. The population size of the herd type is an important index of the prevalence of *Brucella* species. In Iran, the prevalence of *B. melitensis* is higher than *B. abortus* due to the fact that the population of small ruminants is larger than the population of cows [11, 15, 16]. On the other hand, *B. abortus* is the predominant species in Tanzania [17], Uganda [18], and southern Cameroon [19], in which the population of cows is larger than the population of small ruminants. These results stem from the fact that *B. abortus* and *B. melitensis* are the etiological factors of brucellosis in cows and sheep, respectively. Nonetheless, the important point, which causes concern, is the emergence of *Brucella* species in nonspecificity hosts. In the present study, the proportion rate of *B. abortus* in small ruminants was determined to be 8.66%. In comparison with the results from those of other countries including the United States [13], Nigeria [14], and Egypt [6], the present results shows a much higher proportion of *B. abortus* in sheep and goats.

In some areas of Iran, farmers keep herds of cows along with small ruminants in a traditional way to improve economic profit by selling cow milk due mainly to the geographical conditions of these regions (such as having irrigated lands) and the favorable nutritional conditions for keeping livestock. This issue is one of the main factors in the increase in the proportion of Brucellosis in nonspecificity hosts. It was a major issue in Lorestan province. However, in Hamadan province, due to the dryness of the agricultural lands and the inappropriate conditions of livestock feeding, which prevented cattle from being kept alongside small ruminants, it was not a serious problem so that a small number of cows were observed in the farms of small ruminants in the sampling areas of Hamadan province.

Notwithstanding, Aleshtar city, which is located in the north of Lorestan province, has high quality agriculture and takes advantage of irrigated farming. In this city, more than 80% of the farmers keep cattle along with herds of small ruminants in a traditional way. The results of this study showed that this area had the highest incidence of the small ruminants which were infected with *B. abortus*. That is, in this area, 7 of the 13 samples of the identified *Brucella* samples were strains of *B. abortus* (Table 1). A relevant study was conducted in a rural area of Aleshtar region. In this study, 10 samples (5 sheep and 5 goats) were taken from a farm in which 2 members of the family (48-year-old mother and her 23-year-old son) had all of the clinical symptoms of Brucellosis, were diagnosed using 1/640 Wright test, and were treated by a doctor. Based on the results of this study, 3 heads of goats and one head of sheep were infected with *B. abortus*. Moreover, two goats and one sheep were simultaneously infected with *B. abortus* and *B. melitensis*. This result was in line with the results of the study by ZareBidaki et al. [20] in Iran and Wareth et al. [6] in Egypt, who highlighted the fact that a host can be infected with two different species of *Brucella* at the same time. Furthermore, they showed that the effect of livestock contact with the pathogen on the incidence of *B*. *abortus* infection in small ruminants has surpassed the barrier of host specificity. The important point is that goats had a closer contact with the herd of cows on the farm and showed a higher rate of infection with this disease. In addition, there were no cases of abortion among the cow population (i.e., 5 head of cows), in the year of the study. Nonetheless, according to the farmer, one of the cows had an abortion one year before the year of the study which was probably due to brucellosis considering the fact that the family members were infected with this disease. Therefore, one of the main reasons behind the infection of the small ruminants with *B. abortus* is contact with the secretions of infected cows. That is, the spread of *Brucella* from certain hosts to other hosts depends on the breeding conditions of the livestock.

Although *B. abortus* prefers cattle as a specific host, it can also infect sheep, goats, and humans. So, if the prevalence of *B. abortus* in cattle is high in a region, the possibility of sheep and goat infection with this bacterial species may be increased. If control and eradication programs are not carried out continuously in cattle, or if vaccine strains with little protection, such as the *B. abortus* RB51 (RB51) vaccine strain, are used for vaccination in high-prevalence areas, the possibility of small ruminant infection with *B. abortus* will greatly increase. To reduce the possibility of this transmission, regular vaccination of small ruminants and cattle is essential. *B. abortus* S19 (S19) and *B. melitensis* Rev1 (Rev1) vaccines have cross-protective. RB51 is a vaccine strain with very little protection. In areas with a high prevalence of *B. abortus* among cattle, the preferred vaccine is the S19 strain, which should be administered to young heifers on time and regularly [21].

In Iran, *B. abortus* S19 (S19) vaccine was used for cattle vaccination until 2005. However, due to the policies adopted by the Veterinary Organization regarding the interference of this vaccine with the common serological tests of the Iranian Veterinary Organization (impairment in the subtraction of titers caused by vaccination and natural brucellosis infection), the use of the *B. abortus* S19 vaccine was prohibited, and since 2005, full-dose *B. abortus* RB51 vaccine for calves 10 to 34 × 10^9^ CFU and reduced-dose 1 to 3 × 10^9^ CFU have been replaced for cattle vaccination in the whole country. This is while despite the implementation of the program, the desired result has not been achieved and the prevalence of brucellosis in the country is still high, so that in a study conducted in Lorestan province in 2020, from the investigation of the presence of bovine brucellosis among the cattle population, the degree of contamination of milk samples with *Brucella* spp. and *B. abortus* were determined as 26% and 19%, respectively [22]. In Iran, since 2022, Iriba (Iranian Razi Institute *Brucella abortus*) vaccine, which is a modified S19 strain, is used as a full-dose 10 to 34 × 10^9^ CFU in calves and reduced-dose 1 to 3.4 × 10^9^ CFU in cattle. Probably one of the reasons for the higher-than-expected prevalence of *B. abortus* among small ruminants in this study may be due to the policy change from using *B. abortus* S19 vaccine in the vaccination of the cattle population during 17 years (2005–2022) and its subsequent replacement with *B. abortus* RB51 vaccine.

Keeping *Brucella* positive cows with small ruminants is a very important risk factor due to the long life of cows, the high potential of the cows to be asymptomatic vectors in the spread of the disease, and the long duration of the lactation period of the cows. In fact, cows remain in the herd for many years and are more susceptible to *Brucella* infection in comparison with the small ruminants. As a result, the risks of the infection of the cows with the disease and the spread of the disease by these animals are high. Therefore, carrying out annual examinations of the health of the cows which are kept in the vicinity of small ruminants is necessary. The aforementioned examinations are not carried out in Iran. Therefore, this issue can be one of the important reasons behind the increase in *Brucellosis* despite the implementation of the vaccination programs in the annual reports.

## 5. Conclusion

To prevent the continuation of brucellosis in the herd, it is necessary to prevent the simultaneous keeping of cattle next to the herd of small ruminants, and the cows that have a common farm or are close to the place where small ruminants are kept should be tested annually. Also, the protective effect of S19 vaccine (Iriba) in Iran, especially in areas where *B. abortus* contamination of small ruminants has been reported, should be monitored after a few years. On the other hand, abortion in the population of small ruminants due to *B. abortus* should not be ignored and more detailed plans should be made regarding continuous vaccination in sheep, goats, and cattle. Finally, due to the possibility of human contracting brucellosis by consuming raw milk, its public health importance should also be emphasized.

## Figures and Tables

**Table 1:** The number of collected milk samples and the percentage of *Brucella* positive cases based on genus/species and also the sampling location.

Province	City	Livestock *n*		*Brucella*spp. positive (%)	*B. melitensis* (%)	*B. abortus* (%)	*B. melitensis* + *B. abortus* (%)	Keeping cows in the farm
Hamedan	Razan	Sheep	17	6 (35.2)	5 (29.4)	1 (5.8)	0	No
		Goat	7	3 (42.8)	3 (42.8)	0	0	
	Kabodar Ahang	Sheep	15	4 (26.6)	4 (26.6)	0	0	^*∗*^limited
		Goat	11	5 (45.4)	3 (27.2)	2 (18.1)	0	

Lorestan	Khorram Abad	Sheep	22	7 (31.8)	5 (22.7)	2 (9.09)	0	Yes
		Goat	4	0	0	0	0	
	Alashtar	Sheep	16	7 (43.7)	4 (25)	3 (18.7)	1 (6.2)	Yes
		Goat	9	5 (55.5)	1 (11.1)	4 (44.4)	2 (22.2)	
	Azna	Sheep	15	2 (13.3)	2 (13.3)	0	0	No
		Goat	10	3 (30.0)	3 (30)	0	0	
	Kouhdasht	Sheep	17	5 (29.4)	4 (23.5)	1 (5.8)	0	No
		Goat	7	2 (28.5)	2 (28.5)	0	0	

Total		Sheep	102	31 (30.3)	24 (23.5)	7 (6.8)	1 (0.9)	—
		Goat	48	18 (37.5)	12 (25.0)	6 (12.5)	2 (4.1)	—
		Sheep + goat	150	49 (32.6)	36 (24.0)	13 (8.6)	3 (2.0)	—

^*∗*^limited: less than one cow per farm.

## Data Availability

All data are included within the article.
